# Chronic Air Pollution Exposure and the Immunomolecular Profile of Lung Cancer: A Regional Cohort Study

**DOI:** 10.3390/biomedicines14071615

**Published:** 2026-07-17

**Authors:** Denisa-Gabriela Ion-Andrei, Andreea Barde, Cristina Cioti, Izabela Ciofu, Ștefan Dumitrache-Rujinski, Oana Cristina Arghir, Cristian Opariuc-Dan, Elena Danteș

**Affiliations:** 1 Doctoral School of Medicine, “Ovidius” University of Constanta, 900470 Constanta, Romaniaarghir_oana@yahoo.com (O.C.A.);; 2 3rd Department–Clinical Medical Disciplines, Faculty of Medicine, “Ovidius” University of Constanta, 900470 Constanta, Romania; 3 “St. Apostle Andrew” Emergency County Clinical Hospital, 900591 Constanta, Romania; 4 Faculty of Medicine, “Ovidius” University of Constanta, 900470 Constanta, Romania; izabela_elena22@yahoo.com; 5 1st Pulmonology Section, 4th Department—Cardio-Thoracic Pathology, “Carol Davila” University of Medicine and Pharmacy, 020021 Bucharest, Romania; stefan.dumitrache@umfcd.ro; 6 4th Pulmonology Department, “Marius Nasta” Institute of Pneumology, 050159 Bucharest, Romania; 7 Clinical Hospital of Pneumopthisiology Constanta, 900002 Constanta, Romania; 8 Psychology and Educational Sciences, Alexandru Ioan Cuza University, Toma Cozma Street, No. 3, 700553 Iași, Romania

**Keywords:** lung cancer, air pollution, PD-L1, EGFR, environmental exposure, molecular profile

## Abstract

**Background/Objectives:** Air pollution is progressively acknowledged not only as a carcinogenic exposure, but also as a potential modulator of tumor immune activation and molecular heterogeneity in lung cancer. However, integrated analyses combining long-term environmental exposure, molecular biomarkers and immune phenotype remain limited, especially in Eastern European populations. **Methods:** We conducted a regional retrospective study including patients diagnosed with lung cancer in Constanța County, Romania. Prolonged atmospheric pollution exposure over an average period of 15 years was estimated using historic environmental monitoring data. Tumor samples were assessed for histopathological subtype, PD-L1 expression and EGFR status, while ALK and KRAS alterations were descriptively analyzed because of the limited number of positive cases. Multivariate ordinal and binary logistic regression models and predicted probability analyses were used to evaluate the association between chronic pollution exposure, PD-L1 expression and EGFR positivity. **Results:** Chronic NO_2_ exposure was significantly associated with increased PD-L1 expression (OR = 1.035, *p* = 0.017), suggesting an association between NO_2_ exposure and an immune-related tumor phenotype. Similar trends were observed for PM10, NO and NOx exposures. Exploratory associations were observed between EGFR positivity and carbon monoxide exposure. **Conclusions:** Chronic atmospheric pollution was associated with differences in the immune and molecular profile of lung cancer. Long-term exposure to selected pollutants was associated with variations in PD-L1 expression and EGFR status, suggesting that environmental factors may contribute to tumor heterogeneity. However, causal relationships cannot be established from this retrospective observational study.

## 1. Introduction

Lung cancer remains the leading cause of cancer-related death worldwide, despite major advances in molecular diagnostics, targeted therapies and immunotherapy [1]. Although tobacco smoking continues to be the principal risk factor for bronchopulmonary carcinogenesis, increasing evidence suggests that environmental exposures, particularly chronic atmospheric pollution, play an important role not only in tumor initiation, but also in tumor progression, immune modulation and tumor biological behavior [2,3], being classified as a human carcinogen by the International Agency for Research on Cancer (IARC) [4].

The respiratory consequences of chronic exposure to air pollution have become a major global public health concern, particularly in low- and middle-income settings, where preventive environmental interventions remain limited [5,6,7].

Experimental and epidemiological evidence suggests that atmospheric pollution may contribute not only to carcinogenesis itself, but also to the molecular and immunological profile of lung tumors. In recent years, lung cancer biology has increasingly been understood as the result of complex interactions between environmental exposure, chronic inflammation, oxidative stress, molecular susceptibility, and alterations within the tumor microenvironment [8,9,10]. Air pollution has been associated with the expansion of epithelial clones harboring oncogenic driver mutations, including EGFR alterations, possibly accelerating malignant transformation even in non-smokers [8,9]. In parallel, traffic-related pollutants such as NO_2_ and particulate matter have been associated with increased inflammatory signaling, altered immune surveillance, and enhanced programmed death-ligand 1 (PD-L1) expression within the tumor microenvironment [9,11]. These mechanisms could participate in immune escape, tumor aggressiveness, and variability in response to immunotherapy [11].

Lung cancer is currently recognized as a highly heterogeneous disease, distinguished by distinct histopathological, molecular and immunological subtypes. In non-small cell lung cancer (NSCLC), biomarkers such as epidermal growth factor receptor (EGFR) mutations, anaplastic lymphoma kinase (ALK) rearrangements, KRAS mutations and PD-L1 expression play a central role in therapeutic decision making and prognostic stratification [12,13,14].

Despite growing international interest in environmental oncology, important gaps remain in the current literature. Most available studies originate from Asian or North American populations, while integrated regional analyses from Eastern Europe remain scarce. Furthermore, few studies simultaneously evaluate long-term exposure to atmospheric pollution, molecular biomarkers and immune phenotype in lung cancer patients. However, the environmental determinants influencing the distribution of these biomarkers remain insufficiently understood [15].

The Black Sea harbor area of Constanța is a relevant regional model for investigating these associations due to its heterogeneous environmental characteristics, including heavy urban traffic, industrial activity and port related emissions [16]. These conditions produce considerable variability in chronic atmospheric exposure and provide an appropriate framework for assessing the relationship between environmental pollution and lung cancer biology [16].

Accordingly, this analysis investigated the relationship between long-term atmospheric pollution exposure and the immunomolecular landscape of lung cancer in a regional Eastern European cohort.

## 2. Materials and Methods

### 2.1. Study Design and Patient Population

This retrospective observational study initially included 393 patients with histopathologically confirmed lung cancer diagnosed between January 2023 and December 2025 at the Clinical Hospital of Pneumophthisiology, Constanța, Romania. The study cohort represented the overall regional database used for environmental, clinical and molecular investigations. Because biomarker availability and analyzable TPS data varied across patients, inferential analyses were performed on variable-complete-case subgroups, with up to 316 evaluable cases included in PD-L1 ordinal regression analyses and 267 evaluable cases in EGFR logistic regression analyses.

Geographical, clinical, histopathological, immunohistochemical and molecular data were retrospectively collected from medical records and integrated into a real-world regional cohort. Patients were included in the overall cohort if demographic, clinical, histopathological, and residential exposure data were available. Biomarker-specific analyses were restricted to patients with available PD-L1 or EGFR data required for each corresponding model. Patients with incomplete clinical records, unavailable geographic exposure data, or missing PD-L1 or EGFR testing required for the corresponding analyses were excluded from inferential analyses. The study primarily focused on the association between long-term exposure to atmospheric pollution, PD-L1 expression and EGFR status.

### 2.2. Clinical, Histopathological, and Molecular Variables

PD-L1 expression was assessed using the tumor proportion score (TPS) and classified as negative (<1%), low/intermediate (1–49%) or high (≥50%). PD-L1 testing was performed as part of routine clinical practice using validated immunohistochemical assays, including the 22C3 (Dako Agilent Technologies, Santa Clara, CA, USA), SP263 (Ventana Ventana Medical Systems, Tucson, AZ, USA), and SP142 (Ventana) antibody clones. Because this was a retrospective real-world cohort, different assays were used according to institutional diagnostic practice during the study period. PD-L1 assessment was performed predominantly in NSCLC cases, while only a limited number of small-cell lung cancer cases underwent PD-L1 testing as part of routine clinical practice. The EGFR mutation status was evaluated using routine molecular diagnostic techniques according to organizational procedures and was analyzed as a binary variable. Because this was a retrospective real-world cohort, the biomarker availability varied across patients during the study period. ALK rearrangements and KRAS mutations were additionally recorded, but due to the limited number of positive cases, these biomarkers were only descriptively evaluated and were not included in inferential multivariate analyses.

### 2.3. Assessment of Atmospheric Pollution Exposure

Long-term air pollution exposure was retrospectively estimated using historical environmental monitoring data obtained from the Romanian National Air Quality Monitoring Network, publicly available through the national platform calitateaer.ro. Within Constanța County, eight regional air quality monitoring stations (CT-1 to CT-8) were included in the model of exposure assessment. These stations continuously record the annual mean concentrations of major atmospheric pollutants and provide standardized environmental monitoring data for the region.

The pollutants available within the regional monitoring network included carbon monoxide (CO), nitrogen dioxide (NO_2_), nitric oxide (NO), nitrogen oxides (NOx), sulfur dioxide (SO_2_), ozone (O_3_), particulate matter with aerodynamic diameter ≤2.5 μm (PM2.5) and particulate matter with aerodynamic diameter ≤10 μm (PM10), benzene, toluene, M-Xylene, p-xylene, o-xylene, arsenic, cadmium, nickel, and lead. Pollutant concentrations were expressed using the units reported by the Romanian National Air Quality Monitoring Network. NO_2_, NO, NOx, SO_2_, PM10, and PM2.5 concentrations were expressed in μg/m^3^, whereas CO concentrations were expressed in mg/m^3^. The regression coefficients and odds ratios therefore represent the effect associated with a one-unit increase in the respective pollutant concentration. All available pollutants were initially evaluated within the environmental exposure dataset. Several traffic-related pollutants, particularly NO_2_, NO, NOx, and PM10, may share common emission sources and may therefore exhibit substantial correlations. Because the present study employed single-pollutant models and formal correlation analyses among pollutants were not performed, the observed associations should be interpreted cautiously, as they may reflect broader pollution mixtures rather than independent pollutant-specific effects.

For each patient included in the study, the residential location at the time of diagnosis was identified from the official address recorded in the medical documentation. Geographic coordinates (latitude and longitude) were subsequently calculated and each patient was assigned to the nearest air quality monitoring station based on proximity. This approach allowed the retrospective estimation of individual long-term environmental exposure using the nearest available regional pollution measurements.

Average atmospheric pollution exposure was estimated over approximately 15 years before lung cancer diagnosis, consistent with exposure intervals commonly used in environmental oncology and respiratory epidemiology studies evaluating the long-term carcinogenic effects of air pollution. Exposure dynamics were additionally assessed at 3-year intervals throughout the retrospective exposure period in order to account for temporal variations in pollutant concentrations.

The analyzed pollutants were selected based on their established or suspected role in chronic respiratory inflammation, oxidative stress, pulmonary carcinogenesis, immune dysregulation, and tumor progression.

### 2.4. Statistical Analysis

All statistical analyses were performed in R version 4.6 (R Foundation for Statistical Computing, Vienna, Austria; https://www.r-project.org/). Specialized packages for ordinal logistic regression, generalized linear modeling and graphical prediction modeling.

Ordinal logistic (proportional odds) models were estimated using the logit link function, which estimates the cumulative log odds of moving to the higher ordinal category. Estimated cut-points between the ordinal categories were flexible, freely estimated, without constraints.

The accepted models had the maximum gradient size at convergence as close to zero as possible, and, for Hessian conditioning, models with values below 10,000 were admitted; those between 10,000 and 1,000,000 were accepted, but interpretation should be done with caution due to potential collinearity problems.

To assess the models’ explanatory power, pseudo R^2^ values were used, along with their interpretative benchmarks. McFadden R^2^ is the most widely used pseudo-R^2^ for logistic and ordinal models; values above 0.30 indicate an excellent model, and those between 0.10 and 0.30 indicate a very good model. A moderate model is characterized by values between 0.05 and 0.10, and 0.05, the model is weak, on the limit of rejection. Cox–Snell R^2^ generally yields lower values; values above 0.30 indicate a strong model, between 0.15 and 0.30 a moderate model, between 0.05 and 0.15 a weak–moderate model, and below 0.05 a very weak model, on the limit of rejection.

The proportional odds assumption was assessed using the nominal and scale tests from the “ordinal” package. To test for violations of the proportionality assumption for coefficients, the conceptual equivalent of the Brant test for ordinal logistic mode was used under the null hypothesis H0: the predictor coefficients are the same across all categorical thresholds. Therefore, if the ordinal model is correctly specified, the effect of the predictors should be proportional across all transitions. A non-significant test result (*p* > 0.05) indicates that the proportional odds assumption is satisfied. Conversely, a statistically significant result (*p* < 0.05) suggests that the predictors may have disproportionate effects across outcome categories, indicating violation of the standard ordinal model. In such cases, more flexible approaches, such as partial proportional odds models, may be considered.

To test for violation of the assumption of constant error variance, an equivalent of the Brant test was also used for ordinal logistic models, under the null hypothesis H0: the latent variance is constant across all levels of the predictors. If the null hypothesis is rejected, the model has latent heteroscedasticity, meaning that the error variability depends on a predictor, and it is recommended to use a scale effect model; otherwise (*p* > 0.05), the error variance is constant.

Because of the exploratory nature of the environmental analyses and the relatively limited sample size, no formal correction for multiple comparisons was applied. Consequently, marginal associations should be interpreted cautiously and considered hypothesis-generating.

The anonymized study dataset has been deposited in the Open Science Framework (OSF) repository to facilitate transparency and independent evaluation of the reported analyses.

## 3. Results

### 3.1. Patient Characteristics and Tumor Profile

The overall study cohort consisted of 393 patients diagnosed with lung cancer in Constanța County, Romania. Due to incomplete molecular or immunohistochemical testing, the effective number of patients included in inferential analyses varied by biomarker availability and model specification.

The cohort was predominantly male and comprised current or former smokers. NSCLC, especially adenocarcinoma, represented the predominant histopathological subtype, in line with contemporary epidemiological patterns. PD-L1 expression and EGFR mutation status were the principal biomarkers included in inferential analyses, while ALK rearrangements and KRAS mutations were assessed descriptively because of the limited number of positive cases (Table 1).

### 3.2. Association Between Atmospheric Pollution Exposure and PD-L1 Expression Based on TPS

Analysis of the association between atmospheric pollutant exposure and PD-L1 expression highlighted NO_2_ as a statistically significant pollutant, while PM10, NO, and NOx were marginally significant; the remaining pollutants did not demonstrate statistically significant associations.

The analysis of the effect of pollutants on TPS levels was based on a variable number of cases, ranging from 52 to 316. The maximum gradients of the models were very close to zero, with all thresholds stable, suggesting numerical convergence. However, Hessian conditioning values varied considerably across pollutants, and several models showed elevated values, indicating potential instability of parameter estimates and possible collinearity. Therefore, results derived from models with high Hessian conditioning values, especially marginal associations, should be interpreted cautiously and considered exploratory.

Akaike Information Criterion (AIC) values ranged from 116 to 640, indicating a reasonable fit for the model’s complexity. McFadden pseudo-R^2^ values ranged between 0.048 and 0.856, while Cox–Snell pseudo-R^2^ values ranged between 0.076 and 0.757, indicating variable explanatory power across models, from very low explanatory capacity (McFadden pseudo-R^2^ = 4.8%, Cox–Snell pseudo-R^2^ = 7.6%) to very high explanatory capacity (McFadden pseudo-R^2^ = 85.6%, Cox–Snell pseudo-R^2^ = 75.7%). These fit indices should be interpreted together with the Hessian conditioning values, particularly for models with elevated conditioning and limited sample size. However, reduced explanatory power is not uncommon in epidemiological research involving biological and environmental variables (Table 2).

- Our data showed that exposure to NO_2_ was statistically significantly associated with an increased probability of having a higher PD-L1 level, more precisely with a higher PD-L1 expression levels (B = 0.034, OR = 1.035, z = 2.385, *p* = 0.017). Thus, each additional unit of NO_2_ increased the chance of being in a higher PD-L1 category by 3.4%.

The thresholds between PD-L1 categories were significant for the transition to the “high” category, indicating an adequate separation of ordinal levels (B = 3.451, OR = 31.546, z = 3.398, *p* < 0.001), the model suggesting a more difficult transition from the “low/intermediate” category to the “high” category, but not from the “negative” category to the “low/intermediate” category (B = 1.245, OR = 3.474, z = 1.247, *p* = 0.212) (Table 3).

**Figure 1:**
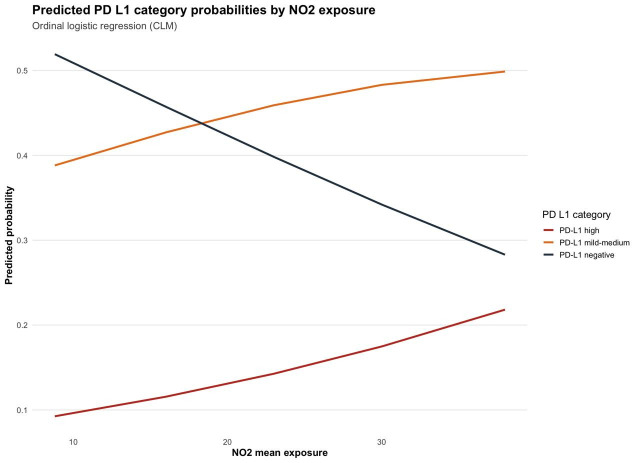
Predicted probabilities of PD-L1 TPS categories according to mean NO_2_ concentration (μg/m^3^). Increasing NO_2_ exposure was associated with a progressive decrease in the probability of PD-L1 negativity, accompanied by modest increases in the probabilities of both low/intermediate and high PD-L1 expression. The most pronounced change was the reduction in the probability of PD-L1-negative tumors, whereas the probability of high PD-L1 expression increased gradually across the exposure range. Overall, the figure suggests a shift away from PD-L1 negativity toward higher PD-L1 expression categories with increasing NO_2_ exposure.

Based on this model for NO_2_ (B = 0.034, *p* = 0.017), a monotonic, consistent positive relationship was observed, indicating that as average exposure to NO_2_ increases, the probability that a patient has high PD-L1 expression also increases significantly (Figure 1).

b. PM 10 exposure also showed a positive, but marginally significant association with PD-L1 levels (B = 0.044, OR = 1.045, z = 1.71, *p* = 0.087). There were no other statistically or marginally significant predictors, but the thresholds between PD-L1 categories were significant for the transition to the “high” category, indicating an appropriate separation of ordinal levels (B = 3.663, OR = 38.973, z = 3.215, *p* = 0.001) and a more difficult transition toward the “high” category.

As the average PM10 exposure increases, the probability of PD-L1 high and PD-L1 mild–medium increases, while the probability of PD-L1 negative decreases (Table 4).

Based on this model for PM10 (B = 0.044, *p* = 0.087), the effect was monotonic, borderline, and visible across all three categories, even though it was only marginally significant overall.

**Figure 2:**
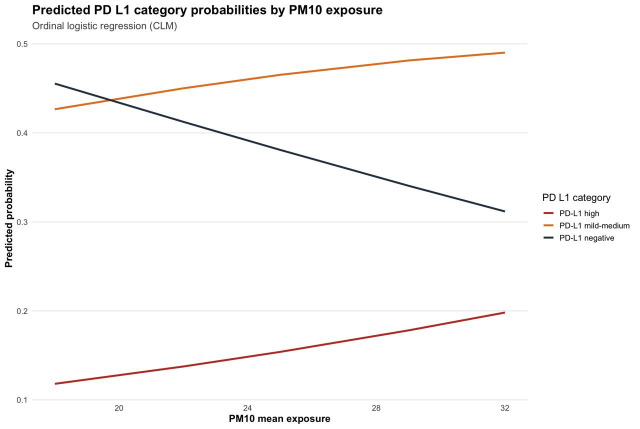
Predicted probabilities of PD-L1 TPS categories according to mean PM10 concentration (μg/m^3^).

Similar trends were observed for PM10 exposure. Increasing PM10 levels were associated with a progressive decrease in the probability of PD-L1 negativity and with increasing probabilities of intermediate and high PD-L1 expression, particularly for the PD-L1 high category, where the ascending trend was most pronounced (Figure 2).

c. NO exposureshowed a marginally significant effect of this pollutant (B = 0.034, OR = 1.035, z = 1.685, *p* = 0.092). There were no other statistically or marginally significant predictors, but the thresholds between PD-L1 categories were significant for the transition to the “high” category, indicating an appropriate separation of ordinal levels (B = 2.974, OR = 19.565, z = 3.058, *p* = 0.002) and a more difficult transition toward the “high” category (Table 5).

**Figure 3:**
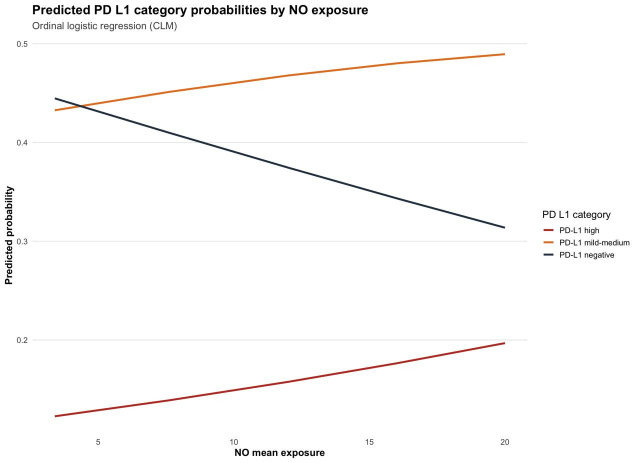
Predicted probabilities of PD-L1 TPS categories according to mean NO concentration (μg/m^3^)**.** Similar exposure-dependent trends were observed for NO. Increasing NO exposure was associated with lower probabilities of PD-L1 negativity and progressively higher probabilities of intermediate and high PD-L1 expression, particularly for the PD-L1 high category.

A monotonic effect was observed throughout the exposure interval, with increasing NO exposure associated with a higher probability of PD-L1 high and PD-L1 mild–medium expression and a lower probability of PD-L1 negative expression (Figure 3).

d. NOx exposureincreased the probability of higher PD-L1 expression by approximately 1% (B = 0.012, OR = 1.012, z = 1.9, *p* = 0.057). There were no other statistically or marginally significant predictors, but the thresholds between PD-L1 categories were significant for the transition to the “high” category, indicating an appropriate separation of ordinal levels (B = 3.11, OR = 22.416, z = 3.157, *p* = 0.002) and a more difficult transition toward the “high” category (Table 6).

**Figure 4:**
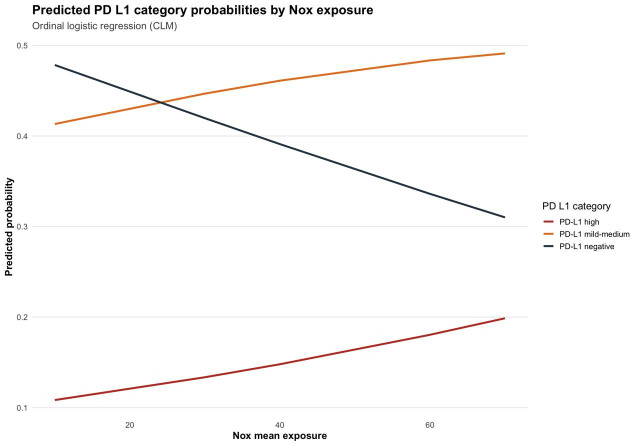
Predicted probabilities of PD-L1 TPS categories according to mean NOx concentration (μg/m^3^). Similar findings were observed for NOx exposure. Increasing NOx levels were associated with lower probabilities of PD-L1 negativity and higher probabilities of intermediate and high PD-L1 expression, with the clearest ascending trend observed in the PD-L1 high category.

It is observed that increasing NOx exposure is associated with a higher probability of PD-L1 high and PD-L1 mild–medium, and a lower probability of PD-L1 negative; the interpretation is identical (Figure 4).

### 3.3. Association Between Atmospheric Pollution Exposure and EGFR Positivity

The analysis of the effect of pollutants on EGFR-positive values was performed using a variable number of cases, ranging from 44 to 267. The models estimated the probability of a patient being EGFR-positive as a function of exposure to pollutants and clinical-demographic characteristics; the only pollutant with a statistically significant effect was CO, to which M-Xylene was added, with a marginal effect.

Overall, the models reduced deviance by 11.141–36.028 points, which in most cases represented moderate improvements. The Akaike Information Criterion was relatively low for a logistic model with nine predictors, ranging from 47.76 to 180.035, thus supporting overall reasonable models.

McFadden pseudo-R^2^ explanatory power values ranged between 0.118 and 0.458, while Cox–Snell pseudo-R^2^ values ranged between 0.082 and 0.219, indicating variable explanatory power across models, from very low (McFadden pseudo-R^2^ = 11.8%, Cox–Snell pseudo-R^2^ = 8.2%) to high explanatory capacity (McFadden pseudo-R^2^ = 45.8%, Cox–Snell pseudo-R^2^ = 21.9%) (Table 7).

CO was the only pollutant reaching statistical significance in the EGFR models (B = 5.865, t = 2.039, *p* = 0.041, OR = 352.339, 95% CI [1.339, 116,191.383]). The association should be interpreted cautiously because of the wide confidence interval, the limited number of EGFR-positive cases, and the original measurement scale of CO concentrations. The large odds ratio therefore reflects the effect associated with a one-unit increase in CO concentration and should not be interpreted as a clinically meaningful effect size. However, the very wide confidence interval indicates substantial uncertainty and imprecision of the effect estimate. Therefore, the association should be interpreted cautiously, particularly given the limited number of EGFR-positive cases included in the analysis (Table 8).

Another pollutant, M-Xylene, was marginally and positively associated with EGFR (B = 0.266, t = 1.755, *p* = 0.079, OR = 1.304, 95% CI [0.965, 1.764]), with individuals exposed to M-Xylene having increased odds of being EGFR-positive (Table 9).

### 3.4. Multivariate Models Including Clinical and Demographic Covariates

To examine the robustness of observed associations, additional multivariate regression models including clinical and demographic covariates were performed. The adjusted analyses included age, sex, smoking status, urban residence, arterial hypertension, diabetes mellitus and COPD.

#### 3.4.1. Adjusted Models for PD-L1 Expression

- NO_2_ exposure. It was noteworthy that arterial hypertension was a strong predictor of elevated PD-L1 expression (B = 1.047, OR = 2.85, z = 4.105, *p* < 0.001), with hypertensive patients having almost 3 times the odds of presenting with high PD-L1 levels. Former smoking status was a marginally significant predictor (B = −0.655, OR = 0.519, z = −1.718, *p* = 0.086). Patients who were former smokers had lower odds of presenting elevated PD-L1 expression. Age, sex, current smoking status, urban residence, and diabetes were not significantly associated with PD-L1 levels (Table 10).
- PM10 exposure. Arterial hypertension was again identified as a strong and independent predictor of increased PD-L1 expression (B = 1.031, OR = 2.804, z = 4.054, *p* < 0.001), with hypertensive patients having almost 3 times the odds of presenting with high PD-L1 levels. Former smoking status was again a marginally significant predictor (B = −0.672, OR = 0.511, z = −1.766, *p* = 0.077). Patients who were former smokers had lower odds of presenting high PD-L1 expression. Exposure to PM10 was associated with a slight increase in the probability of presenting elevated PD-L1 levels (Table 11).
- NO exposure. A statistically significant effect of arterial hypertension was observed (B = 1.014, OR = 2.757, z = 3.998, *p* < 0.001), together with a marginally significant effect of former smoking status (B = −0.665, OR = 0.514, z = −1.747, *p* = 0.081). Similar to the other pollutants, NO exposure was associated with an increased probability of high PD-L1 expression, while arterial hypertension increased the odds of elevated PD-L1 levels by approximately threefold. In contrast, former smoking status was associated with lower odds of presenting high PD-L1 expression (Table 12).
- NOx exposure. Arterial hypertension was associated with approximately threefold higher odds of elevated PD-L1 expression (B = 1.022, OR = 2.778, z = 4.022, *p* < 0.001), while former smoking status was associated with lower odds of presenting high PD-L1 levels (B = −0.657, OR = 0.518, z = −1.724, *p* = 0.085). Except for arterial hypertension, the other predictors showed only marginal statistical significance (Table 13).

#### 3.4.2. Adjusted Models for EGFR Positivity

- EGFR and CO. Another statistically significant predictor associated with EGFR positivity in the presence of CO exposure was sex (B = 1.858, t = 3.885, *p* < 0.001, OR = 6.408, 95% CI [2.573, 17.06]), the effect being strong and robust, with men having approximately six times higher odds of being EGFR-positive compared to women. Former smoking status was also significantly associated with EGFR positivity (B = 1.741, t = 2.143, *p* = 0.032, OR = 5.702, 95% CI [1.275, 32.778]), with former smokers having approximately five times higher odds of being EGFR-positive compared to non-smokers.

Along with these statistically significant effects, a positive borderline association was observed for current smoking status (B = 1.455, t = 1.89, *p* = 0.059, OR = 4.284, 95% CI [1.049, 22.91]), suggesting that current smokers may have a higher odds of EGFR positivity than non-smokers. In contrast, COPD showed a marginal negative association with EGFR positivity (B = −0.904, t = −1.865, *p* = 0.062, OR = 0.405, 95% CI [0.151, 1.025]), indicating lower odds of EGFR positivity in patients with COPD (Table 14).

**Figure 5:**
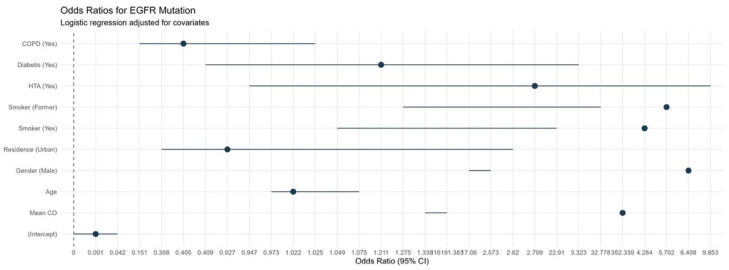
Odds ratios for EGFR positivity according to mean CO concentration (mg/m^3^). Points represent odds ratio estimates and horizontal lines indicate 95% confidence intervals, also the dashed vertical line represents the null value (OR = 1). In the logistic regression model, mean CO exposure was significantly positively associated with EGFR mutation status. Male was significantly associated with higher odds of EGFR positivity compared to female, while former smokers also demonstrated significantly higher odds of EGFR positivity compared to non-smokers. Marginal associations were additionally observed for current smoking status, arterial hypertension, and COPD, although these did not reach statistical significance at *p* < 0.05.

The adjusted odds ratios and confidence intervals for the variables included in the model are illustrated in Figure 5.

b. EGFR and M-Xylene. Similarly, statistically significant positive associations were observed with biological sex (B = 1.453, t = 2.529, *p* = 0.011, OR = 4.276, 95% CI [1.419, 13.858]), with men having approximately four times higher odds of being EGFR-positive compared to women. A marginal positive association was also observed for former smoking status (B = 1.709, t = 1.895, *p* = 0.058, OR = 5.525, 95% CI [1.047, 38.455]), suggesting that former smokers may have a higher odds of EGFR positivity than non-smokers.

In contrast, COPD showed a marginal negative association with EGFR positivity (B = −1.087, t = −1.862, *p* = 0.063, OR = 0.337, 95% CI [0.1, 1.02]), indicating lower odds of EGFR positivity in patients with COPD (Table 15).

**Figure 6:**
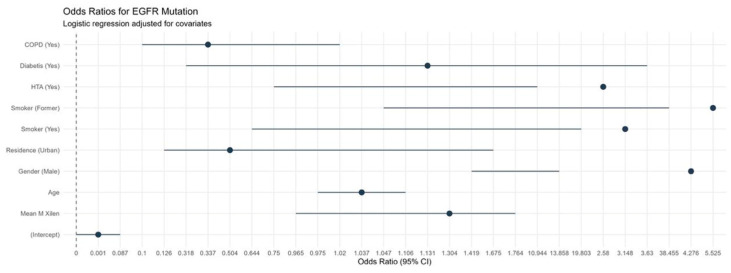
Odds ratios for EGFR positivity according to mean M-xylene concentration (μg/m^3^). Points represent odds ratio estimates and horizontal lines indicate 95% confidence intervals. The dashed vertical line represents the null value (OR = 1). Male was significantly associated with the presence of EGFR mutations, while exposure to M-Xylene demonstrated a positive but marginal trend. Similar marginal associations were also observed for former smoking status and COPD, although these did not reach the threshold for statistical significance. Age, urban residence, current smoking status, arterial hypertension, and diabetes were not significantly associated with EGFR mutation status.

The adjusted odds ratios and confidence intervals for the variables included in the model are illustrated in Figure 6.

## 4. Discussion

This regional study represents one of the first integrated analyses from Eastern Europe evaluating the relationships between chronic atmospheric pollution exposure, PD-L1 expression and EGFR status in lung cancer patients. Through integrating environmental exposure assessment with molecular, immunological and clinical data, our evidence suggests that chronic atmospheric pollution may contribute not only to carcinogenesis, but also to immune modulation and molecular heterogeneity within the tumor microenvironment. Although previous studies have investigated isolated associations between air pollution and oncogenic driver mutations or immune biomarkers, integrated analyses simultaneously evaluating environmental exposure, molecular alterations and immune phenotype remain limited, particularly in Eastern European populations [17].

### 4.1. Air Pollution and PD-L1 Expression

Our study showed significant associations between long-term exposure to traffic-related pollutants and increased expression of PD-L1 in lung cancer patients from Constanța County, Romania. Among the analyzed pollutants, NO_2_ exposure showed the strongest association with elevated PD-L1 levels, while PM10, NO and NOx showed similar borderline trends. These observations support the hypothesis that chronic exposure to traffic and combustion-related pollutants drives a more inflamed and immunologically active tumor microenvironment.

Our results are consistent with the growing body of evidence linking atmospheric pollution to immune modulation in lung cancer. Tamayo et al. reported that NO_2_ exposure was associated with increased PD-L1 expression in patients with non-small cell lung cancer from Bogotá, supporting the hypothesis that traffic-related pollution may contribute to pollution-induced immune dysregulation within lung tumors [15,18]. The concordance between their findings and ours is particularly relevant because both studies integrated environmental exposure with molecular and immune biomarkers despite being conducted in different geographic and ethnic populations.

Several experimental studies may help explain these observations by demonstrating that atmospheric pollutants induce chronic inflammation, oxidative stress, macrophage recruitment, and cytokine release, mechanisms that can promote pro-inflammatory immune activation and tumor immune escape [18]. Chronic inflammatory remodeling and persistent immune dysregulation within the pulmonary microenvironment have also been implicated in other inflammation-associated oncogenic pathways in lung cancer [19]. Persistent exposure to pollutants such as NO_2_, NOx, and particulate matter may therefore contribute to PD-L1 upregulation via chronic inflammatory signaling pathways, potentially favoring a tumor microenvironment better able to suppress cytotoxic T-cell activity.

Notably, several pollution-related exposures displayed consistent directional associations with increased PD-L1 expression across multiple environmental exposure models, even when statistical significance remained borderline. This consistency of directional trends strengthens the biological plausibility of observed associations and supports the hypothesis that chronic atmospheric exposure may cumulatively promote an immunologically permissive tumor phenotype.

### 4.2. Air Pollution and EGFR Positivity

Another exploratory finding from our study was the association between chronic CO exposure and EGFR positivity. However, this association should be interpreted cautiously because the model produced a very wide confidence interval, indicating substantial uncertainty and limited precision of the effect estimate.

Our findings represent exploratory associations that are consistent with emerging evidence suggesting that chronic air pollution may influence the molecular profile of lung cancer. Hill et al. demonstrated that PM2.5 exposure may promote lung adenocarcinoma progression by expanding epithelial clones harboring pre-existing EGFR mutations via IL-1-mediated inflammatory signaling [9]. Tamayo et al. also reported independent associations between long-term exposure to PM2.5 and PM10 and EGFR-mutated NSCLC [15]. Although the specific pollutants associated with EGFR positivity varied across cohorts, these studies collectively support the hypothesis that chronic environmental exposure may contribute to molecularly defined lung cancer phenotypes. Nevertheless, the limited number of EGFR-positive cases and the wide confidence interval observed in the present study suggest that these findings require validation in larger cohorts. The association between male sex, smoking history, and EGFR positivity observed in our adjusted models differs from the classical epidemiological profile of EGFR-mutated NSCLC, which is more frequently reported among women and never-smokers. Several factors may explain this discrepancy. The number of EGFR-positive cases in our cohort was limited, and the study population was predominantly composed of male patients and current or former smokers, reflecting the regional epidemiology of lung cancer. Furthermore, detailed information regarding EGFR mutation subtypes and cumulative smoking exposure (pack-years) was not consistently available, and the limited number of EGFR-positive cases precluded robust stratified analyses according to histology or smoking status. Therefore, these findings should be interpreted cautiously and considered exploratory rather than indicative of a different biological pattern of EGFR-mutated lung cancer.

The relationship between atmospheric pollution and EGFR-positive lung cancer may be particularly relevant in adenocarcinoma and in patients with lower tobacco-related exposure, where ecological carcinogenic mechanisms may contribute more significantly to tumor molecular heterogeneity. However, because our cohort predominantly included male smokers and the number of EGFR-positive cases was limited, these observations should be considered hypothesis-generating. Further studies including detailed smoking exposure, histological stratification, and EGFR mutation subtypes are required to clarify the potential interaction between atmospheric pollution and EGFR-driven lung carcinogenesis. Because of the limited number of positive cases, ALK rearrangements and KRAS mutations could not be included in reliable inferential analyses and were evaluated descriptively only. This limitation is relatively common in retrospective regional cohorts, particularly when molecular testing availability varies over time. Larger molecularly characterized cohorts are therefore needed to clarify whether these biomarkers may also be influenced by chronic environmental exposure.

Overall, these observations suggest that chronic atmospheric pollution exposure may be associated with differences in the distribution of molecular phenotypes. However, these findings remain exploratory and should be confirmed in larger studies with standardized exposure assessment and comprehensive molecular characterization. This concept may be especially relevant in regions defined by long-term exposure to heterogeneous environmental pollutants.

### 4.3. Clinical and Demographic Covariates Associated with Immune and Molecular Profiles

Beyond the direct associations observed between atmospheric pollutants and immune or molecular tumor characteristics, several clinical and demographic covariates demonstrated recurrent significant or marginal associations across multiple environmental exposure models.

Regarding PD-L1 expression, the models suggested that both pollution exposure and cardiovascular comorbidities may partially explain a tumor immune microenvironment characterized by increased PD-L1 expression. In all other models, even when pollutants did not predict PD-L1 levels at the 5% or 10% significance levels, arterial hypertension remained positively and statistically significantly associated with PD-L1 expression (*p* < 0.05). The consistent association between arterial hypertension and increased PD-L1 expression is less directly documented in the literature on lung cancer, but it may be biologically plausible through common pathways involving endothelial dysfunction, chronic vascular inflammation, oxidative stress and immune activation. Therefore, this finding should be considered exploratory and hypothesis generating [20,21,22].

Additional associations were also observed for smoking status and *sex* across several pollutant exposure models. Former smoking status was negatively associated with PD-L1 levels in the presence of SO2 (*p* = 0.077), CO (*p* = 0.093) and O3 (*p* = 0.058). Previous studies evaluating smoking exposure and PD-L1 expression in NSCLC reported heterogeneous results, with certain studies suggesting higher PD-L1 expression among current smokers, likely related to smoking-induced inflammatory and mutational burden [23,24,25,26].

Sex was negatively and statistically significantly associated with PD-L1 levels in the presence of benzene (*p* = 0.043), ethylbenzene (*p* = 0.041), M-Xylene (*p* = 0.028), and As10 (*p* = 0.04), as well as marginally significantly associated in the presence of O-xylene (*p* = 0.052), Cd10 (*p* = 0.089), and Ni10 (*p* = 0.089). Thus, the negative associations suggest that men exposed to these pollutants tended to present lower PD-L1 levels, even when the pollutants themselves were not significant predictors. Data regarding sex-related differences in PD-L1 expression remain inconsistent in the literature, suggesting that hormonal, inflammatory, and environmental factors may variably influence tumor immune phenotypes across different populations [27,28,29].

Additional exploratory associations were observed for diabetes and age in selected pollutant exposure models. In the presence of toluene, a marginal negative association with diabetes was observed (*p* = 0.097), suggesting that diabetic patients tended to have lower PD-L1 levels. The observed negative association between diabetes and PD-L1 expression should be interpreted cautiously, as the available literature regarding the interaction between metabolic disorders and immune checkpoint expression in lung cancer remains limited and inconsistent. In the presence of As10, a borderline-positive association with age was observed (*p* = 0.08), suggesting that older age was associated with higher PD-L1 levels. *Age*-related immune remodeling and chronic low-grade inflammation have previously been proposed as potential contributors to altered immune checkpoint expression in elderly cancer populations, although available evidence in NSCLC remains limited [30].

Regarding EGFR positivity, *COPD* demonstrated recurrent negative associations across multiple environmental exposure models, suggesting that EGFR-mutated phenotypes may occur more frequently in patients without advanced smoking-related chronic pulmonary disease. More precisely, the probability of obtaining positive EGFR values tended to be higher in the absence of COPD, regardless of pollutant significance.

This observation is consistent with previous reports showing a lower prevalence of EGFR mutations among patients with COPD-related NSCLC compared with those without COPD, suggesting that COPD-associated lung cancer may follow different molecular pathways, more closely related to smoking-related airway injury and chronic obstructive remodeling. Lim et al. reported that patients with COPD-related NSCLC had lower prevalences of EGFR mutations and ALK rearrangements, and that this decrease was proportional to the severity of airflow limitation [31].

Male was statistically significantly associated with EGFR positivity across all pollutants, regardless of whether the pollutants themselves showed both statistically significant and marginal associations. The recurrent association between male and EGFR positivity in our models differs from much of the published literature, which reports EGFR mutations more frequently in women, especially in adenocarcinoma and never-smoker populations. This difference may reflect the specific composition of our cohort, regional exposure patterns, smoking distribution, or the interaction between sex, environmental exposure, and tumor molecular phenotype [32,33].

Positive, significant, or marginal associations were also observed for *smoking status*, either current or former. Regardless of the statistical significance of the pollutant, current and former smokers had higher odds of EGFR positivity than non-smokers. The association between smoking status and EGFR positivity should be interpreted cautiously, as most previous studies have reported higher EGFR mutation frequencies among never-smokers compared with smokers. However, EGFR mutations may still occur in former or current smokers, and some studies suggest that mutation patterns may differ according to smoking exposure intensity and mutation subtype [34,35,36].

Additional exploratory associations were also observed between systemic comorbidities and EGFR positivity across several environmental exposure models, suggesting that host-related inflammatory and metabolic factors may further modulate molecular tumor phenotypes in the context of chronic atmospheric exposure. In some cases, such as in the presence of SO_2_ (*p* = 0.062), PM10 (*p* = 0.079), NO (*p* = 0.081), NOx (*p* = 0.08), As10 (*p* = 0.033), Cd10 (*p* = 0.035), Ni10 (*p* = 0.024), and Pb10 (*p* = 0.06), positive statistically significant or marginal associations were observed between arterial hypertension and EGFR positivity. In the presence of ozone, positive associations with diabetes were also observed (*p* = 0.039).

### 4.4. Strengths and Limitations

This study represents one of the first regional real-world analyses from Eastern Europe to evaluate the relationship between chronic atmospheric pollution exposure, PD-L1 expression and EGFR status in lung cancer patients. A major strength of the study is the combination of the environmental exposure assessment with molecular, immunological and clinical data within the same cohort.

Several limitations should also be recognized. First, the retrospective observational design precludes causal inference. Second, formal correlation analyses among pollutants were not performed. Traffic-related pollutants may exhibit substantial correlations because of shared emission sources, and the use of single-pollutant models may not fully disentangle independent pollutant-specific effects. Additionally, several regression models demonstrated elevated Hessian conditioning values, suggesting potential instability of some parameter estimates and reduced reliability of marginal associations. Therefore, pollutant-specific findings, particularly those with borderline statistical significance, should be interpreted cautiously. Third, environmental exposure was estimated using residential proximity to monitoring stations, which may not fully capture individual exposure patterns.

Additional limitations include the limited number of ALK- and KRAS-positive cases, which may have contributed to unstable estimates and wide confidence intervals, particularly for the CO model. Because several environmental exposure analyses were exploratory, and multiple pollutant-endpoint associations were examined, no formal correction for multiple comparisons was performed. Therefore, marginal findings should be interpreted cautiously and require validation in independent cohorts.

### 4.5. Clinical Consequences and Future Perspectives

Overall, these data support the hypothesis that chronic atmospheric pollution contributes not only to lung carcinogenesis, but also to the immune and molecular heterogeneity of lung tumors. The observed associations between traffic and combustion-related pollutants, PD-L1 expression and EGFR positivity suggest that long-term environmental exposure may influence biologically relevant tumor phenotypes.

These observations highlight the need for larger prospective studies that integrate environmental, molecular, immunological and therapeutic data. Incorporation of environmental exposure assessment into thoracic oncology research may improve risk stratification and contribute to a more complete understanding of lung cancer heterogeneity in actual populations.

## 5. Conclusions

This study suggests that long-term exposure to atmospheric pollution should be associated with differences in the immune and molecular profile of lung cancer, supporting its potential role as a biologically relevant contributor to tumor heterogeneity and disease evolution. Chronic exposure to traffic- and combustion-related pollutants, particularly NO_2_, was associated with increased PD-L1 expression, while CO exposure was associated with EGFR positivity. These findings support the hypothesis that environmental exposure may influence the biological behavior and molecular profile of lung tumors. Future longitudinal studies should further investigate these associations and clarify the biological mechanisms linking chronic atmospheric pollution with tumor immune and molecular profiles.

## Figures and Tables

**Table 1:** Baseline clinical, histopathological, and molecular characteristics of the study cohort. Continuous variables are presented as mean ± standard deviation and categorical variables as n (%). Comparisons were performed using Student’s *t*-test, Chi-square test, or Fisher’s exact test, as appropriate. Abbreviations: SD, standard deviation; PD-L1, programmed death-ligand 1; EGFR, epidermal growth factor receptor; ALK, anaplastic lymphoma kinase; KRAS, Kirsten rat sarcoma viral oncogene homolog; NSCLC, non-small cell lung cancer; NOS, not otherwise specified.

	Total Cohort (No, %)	Deceased (No, %)	Survivors (No, %)	*p*-Value
Age, mean ± SD	67.0 ± 9.07	67.4 ± 9.19	66.1 ± 8.74	0.188
Gender				0.311
Female	280 (71.2%)	78 (67.2%)	202 (72.9%)	
Male	113 (28.8%)	38 (32.8%)	75 (27.1%)	
Residence				1.000
Rural	242 (61.7%)	71 (61.7%)	171 (61.7%)	
Urban	150 (38.3%)	44 (38.3%)	106 (38.3%)	
Smoking status				0.730
Current smoker	212 (53.9%)	59 (50.9%)	153 (55.2%)	
Former smoker	130 (33.1%)	41 (35.3%)	89 (32.1%)	
Non-smoker	51 (13.0%)	16 (13.8%)	35 (12.6%)	
Histopathological subtype				0.161
Adenocarcinoma	182 (46.3%)	62 (54.4%)	120 (43.3%)	
Squamous carcinoma	133 (33.8%)	32 (28.1%)	101 (36.5%)	
Small-cell lung carcinoma (SCLC)	58 (14.8%)	17 (14.9%)	41 (14.8%)	
NSCLC NOS/undifferentiated carcinoma	18 (4.6%)	3 (2.6%)	15 (5.4%)	
Immunological and molecular profile				
PD-L1 negative	123 (31.3%)	37 (31.9%)	86 (31.1%)	
PD-L1 low/intermediate	140 (35.6%)	38 (32.8%)	102 (36.8%)	0.726
PD-L1 high	53 (13.5%)	17 (14.7%)	36 (13.0%)	
EGFR positive	30 (7.6%)	12 (10.3%)	18 (6.5%)	0.213
ALK positive	3 (0.8%)	1 (0.9%)	2 (0.7%)	1.000
KRAS positive	3 (0.8%)	3 (2.6%)	0 (0.0%)	0.025

**Table 2:** Model fit statistics of ordinal logistic regression models assessing the association between atmospheric pollutant exposure and PD-L1 TPS categories. Abbreviations: N, number of evaluable cases; TPS, tumor proportion score; Log-Lik., log-likelihood; AIC, Akaike Information Criterion; Max. grad., maximum gradient at convergence; Hessian cond., Hessian conditioning value; McFadden R^2^, McFadden pseudo coefficient of determination; Cox–Snell pseudo-R^2^, Cox–Snell pseudo coefficient of determination.

Pollutant	N	Log-Lik.	AIC	Max. Grad.	Hessian Cond.	McFadden R^2^	Cox–Snell Pseudo-R^2^
NO_2_ (μg/m^3^)	316	−308	637	0	9.2 × 10^5^	0.05	0.08
SO_2_ (μg/m^3^)	291	−285	592	0	9.0 × 10^5^	0.12	0.18
PM10 (μg/m^3^)	316	−309	640	0	1.1 × 10^6^	0.05	0.08
CO (mg/m^3^)	291	−285	591	0	1.0 × 10^6^	0.12	0.18
NO (μg/m^3^)	316	−309	640	0	7.7 × 10^5^	0.05	0.08
NOx (μg/m^3^)	316	−309	639	0	1.1 × 10^6^	0.05	0.08
Benzene (μg/m^3^)	185	−183	388	0	7.1 × 10^5^	0.44	0.51
Ethylbenzene (μg/m^3^)	193	−191	405	0	6.9 × 10^5^	0.41	0.49
M-Xylene (μg/m^3^)	193	−191	404	0	7.0 × 10^5^	0.41	0.49
O-Xylene (μg/m^3^)	200	−199	421	0	6.7 × 10^5^	0.39	0.47
P-Xylene (μg/m^3^)	214	−215	452	0	7.3 × 10^5^	0.34	0.43
Toluene (μg/m^3^)	165	−164	351	0	8.6 × 10^5^	0.49	0.56
As (ng/m^3^)	10	−154	330	0	8.2 × 10^5^	0.53	0.58
Cd (ng/m^3^)	10	−174	369	0	3.2 × 10^6^	0.47	0.54
Ni (ng/m^3^)	10	−173	369	0	9.2 × 10^6^	0.47	0.54
Pb (ng/m^3^)	10	−209	440	0	8.4 × 10^7^	0.36	0.44
O_3_ (μg/m^3^)	172	−164	351	0	2.4 × 10^6^	0.49	0.56
PM2.5 (μg/m^3^)	52	−47	116	0	2.7 × 10^6^	0.86	0.76

**Table 3:** Ordinal logistic regression model evaluating the association between mean NO_2_ exposure and PD-L1 TPS categories. Higher NO_2_ exposure was associated with increased odds of belonging to a higher PD-L1 expression category. Abbreviations: NO_2_, nitrogen dioxide; TPS, tumor proportion score; B, regression coefficients; OR, odds ratio; SE, standard error.

Variable	B	OR	SE	z-Value	*p*-Value
PD-L1 negative → PD-L1 “low/intermediate”	1.245	3.474	1.00	1.247	0.212
PD-L1 “low/intermediate” → PD-L1 high	3.451	31.546	1.02	3.398	<0.001
Mean NO_2_ (μg/m^3^)	0.034	1.035	0.01	2.385	0.017

**Table 4:** Ordinal logistic regression model evaluating the association between mean PM10 exposure and PD-L1 TPS categories. Regression coefficients (B), odds ratios (OR), standard errors (SE), z-values, and *p*-values are presented. Abbreviations: PM10, particulate matter with an aerodynamic diameter ≤ 10 μm; TPS, tumor proportion score; OR, odds ratio; SE, standard error.

Variable	B	OR	SE	z-Value	*p*-Value
PD-L1 negative → PD-L1 “low/intermediate”	1.47	4.36	1.12	1.31	0.190
PD-L1 “low/intermediate”→ PD-L1high	3.663	38.973	1.14	3.215	0.001
Mean PM10 (μg/m^3^)	0.044	1.045	0.03	1.71	0.087

**Table 5:** Ordinal logistic regression analysis of mean NO exposure and PD-L1 TPS categories. Abbreviations: NO, nitric oxide; TPS, tumor proportion score; OR, odds ratio; SE, standard error.

Variable	B	OR	SE	z-Value	*p*-Value
PD-L1 negative → PD-L1 “low/intermediate”	0.78	2.19	0.96	0.82	0.414
PD-L1 “low/intermediate”→ PD-L1 high	2.974	19.565	0.97	3.058	0.002
Mean NO (μg/m^3^)	0.034	1.035	0.02	1.685	0.092

**Table 6:** Ordinal logistic regression analysis of mean NOx exposure and PD-L1 TPS categories. Abbreviations: NOx, nitrogen oxides; TPS, tumor proportion score; OR, odds ratio; SE, standard error.

Variable	B	OR	SE	z-Value	*p*-Value
PD-L1 negative → PD-L1 “low/intermediate”	0.92	2.50	0.97	0.94	0.345
PD-L1 “low/intermediate”→ PD-L1 high	3.11	22.416	0.98	3.157	0.002
Mean NOx (μg/m^3^)	0.012	1.012	0.01	1.90	0.057

**Table 7:** Model fit statistics of logistic regression models evaluating the association between atmospheric pollutant exposure and EGFR positivity. Abbreviations: EGFR, epidermal growth factor receptor; N, number of evaluable cases; G^2^ deviance, likelihood-ratio deviance statistic; AIC, Akaike Information Criterion; McFadden R^2^, McFadden pseudo coefficient of determination; Cox–Snell pseudo-R^2^, Cox–Snell pseudo coefficient of determination; Nagelkerke pseudo-R^2^, Nagelkerke pseudo coefficient of determination.

Pollutant	N	G2 Deviance	AIC	McFadden Pseudo-R^2^	Cox–Snell Pseudo-R^2^	Nagelkerke Pseudo-R^2^
NO_2_ (μg/m^3^)	267	27.62	180.04	0.15	0.10	0.20
SO_2_ (μg/m^3^)	246	30.80	159.66	0.18	0.12	0.23
PM10 (μg/m^3^)	267	28.11	179.54	0.15	0.10	0.20
CO (mg/m^3^)	246	32.39	158.07	0.19	0.12	0.25
NO (μg/m^3^)	267	28.61	179.05	0.15	0.10	0.20
NOx (μg/m^3^)	267	28.19	179.47	0.15	0.10	0.20
Benzene (μg/m^3^)	158	13.69	122.68	0.12	0.08	0.16
Ethylbenzene (μg/m^3^)	165	15.45	122.65	0.13	0.09	0.17
M-Xylene (μg/m^3^)	165	18.46	119.64	0.16	0.10	0.21
O-Xylene (μg/m^3^)	168	18.19	116.44	0.16	0.10	0.21
P-Xylene (μg/m^3^)	181	18.25	131.93	0.14	0.10	0.19
Toluene (μg/m^3^)	145	12.48	85.55	0.16	0.08	0.20
As (ng/m^3^)	141	25.61	106.16	0.23	0.16	0.30
Cd (ng/m^3^)	163	28.51	117.00	0.23	0.16	0.30
Ni (ng/m^3^)	163	29.15	116.36	0.23	0.16	0.30
Pb (ng/m^3^)	188	25.32	130.64	0.19	0.12	0.24
O_3_ (μg/m^3^)	149	36.03	62.62	0.46	0.21	0.52
PM2.5 (μg/m^3^)	44	11.14	47.76	0.29	0.22	0.38

**Table 8:** Logistic regression analysis of mean CO exposure and EGFR positivity. Abbreviations: CO, carbon monoxide; EGFR, epidermal growth factor receptor; OR, odds ratio; CI, confidence interval; SE, standard error.

Variable	B	SE	t	*p*-Value	OR	95% CI Lower	95% CI Upper
(Intercept)	−7.286	2.186	−3.334	<0.001	0.001	0	0.042
Mean CO (mg/m^3^)	5.865	2.876	2.039	0.041	352.339	1.339	116,191.383

**Table 9:** Logistic regression analysis of mean M-Xylene exposure and EGFR positivity. Abbreviations: EGFR, epidermal growth factor receptor; OR, odds ratio; CI, confidence interval; SE, standard error.

Variable	B	SE	t	*p*-Value	OR	95% CI Lower	95% CI Upper
(Intercept)	−7.218	2.562	−2.818	0.005	0.001	0	0.087
Mean M-Xylene (μg/m^3^)	0.266	0.151	1.755	0.079	1.304	0.965	1.764

**Table 10:** Clinical and demographic covariates included in the ordinal logistic regression model for PD-L1 TPS categories. Abbreviations: PD-L1, programmed death-ligand 1; TPS, tumor proportion score; OR, odds ratio; SE, standard error; HTA, arterial hypertension; COPD, chronic obstructive pulmonary disease.

Variable	B	OR	SE	z-Value	*p*-Value
Mean NO_2_ (μg/m^3^)	0.03	1.03	0.01	2.38	0.017
Age	0.01	1.01	0.01	0.59	0.556
Male	−0.08	0.92	0.25	−0.33	0.741
Urban residence	0.20	1.22	0.25	0.80	0.426
Current smoker	−0.25	0.78	0.37	−0.69	0.490
Former smoker	−0.655	0.519	0.38	−1.718	0.086
Arterial hypertension	1.047	2.85	0.26	4.105	<0.001
Diabetes mellitus	−0.32	0.73	0.30	−1.07	0.284
COPD	0.09	1.10	0.23	0.41	0.685

**Table 11:** Clinical and demographic covariates included in the ordinal logistic regression model for PM10 and PD-L1 TPS categories. Abbreviations: PD-L1, programmed death-ligand 1; TPS, tumor proportion score; OR, odds ratio; SE, standard error; HTA, arterial hypertension; COPD, chronic obstructive pulmonary disease; PM10, particulate matter with an aerodynamic diameter ≤10 μm.

Variable	B	OR	SE	z-Value	*p*-Value
Mean PM10 (μg/m^3^)	0.04	1.04	0.03	1.71	0.087
Age	0.01	1.01	0.01	0.62	0.537
Male	−0.06	0.95	0.25	−0.23	0.821
Urban residence	0.15	1.16	0.25	0.61	0.540
Current smoker	−0.26	0.77	0.37	−0.70	0.482
Former smoker	−0.672	0.511	0.38	−1.766	0.077
Arterial hypertension	1.031	2.804	0.25	4.054	<0.001
Diabetes mellitus	−0.31	0.73	0.30	−1.05	0.293
COPD	0.09	1.10	0.23	0.42	0.676

**Table 12:** Clinical and demographic covariates included in the ordinal logistic regression model for NO and PD-L1 TPS categories. Abbreviations: PD-L1, programmed death-ligand 1; TPS, tumor proportion score; OR, odds ratio; SE, standard error; HTA, arterial hypertension; COPD, chronic obstructive pulmonary disease; NO, nitric oxide.

Variable	B	OR	SE	z-Value	*p*-Value
Mean NO (μg/m^3^)	0.03	1.03	0.02	1.69	0.092
Age	0.01	1.01	0.01	0.65	0.516
Male	−0.06	0.94	0.25	−0.24	0.811
Urban residence	0.16	1.17	0.25	0.64	0.522
Current smoker	−0.26	0.77	0.37	−0.70	0.486
Former smoker	−0.665	0.514	0.38	−1.747	0.081
Arterial hypertension	1.014	2.757	0.25	3.998	<0.001
Diabetes mellitus	−0.31	0.73	0.30	−1.04	0.297
COPD	0.10	1.11	0.23	0.47	0.642

**Table 13:** Clinical and demographic covariates included in the ordinal logistic regression model for NOx and PD-L1 TPS categories. Abbreviations: PD-L1, programmed death-ligand 1; TPS, tumor proportion score; OR, odds ratio; SE, standard error; HTA, arterial hypertension; COPD, chronic obstructive pulmonary disease; NOx, nitrogen oxides.

Variable	B	OR	SE	z-Value	*p*-Value
Mean NOx (μg/m^3^)	0.01	1.01	0.01	1.90	0.057
Age	0.01	1.01	0.01	0.62	0.532
Male	−0.06	0.94	0.25	−0.25	0.802
Urban residence	0.18	1.20	0.25	0.72	0.473
Current smoker	−0.25	0.78	0.37	−0.69	0.492
Former smoker	−0.657	0.518	0.38	−1.724	0.085
Arterial hypertension	1.022	2.778	0.25	4.022	<0.001
Diabetes mellitus	−0.31	0.73	0.30	−1.05	0.295
COPD	0.10	1.11	0.23	0.45	0.651

**Table 14:** Clinical and demographic covariates included in the multivariate logistic regression model for CO exposure and EGFR positivity. Abbreviations: EGFR, epidermal growth factor receptor; OR, odds ratio; CI, confidence interval; SE, standard error; HTA, arterial hypertension; COPD, chronic obstructive pulmonary disease; CO, carbon monoxide.

Variable	B	SE	t	*p*-Value	OR	95% CI Lower	95% CI Upper
Mean CO (mg/m^3^)	5.865	2.876	2.039	0.041	352.339	1.339	116,191.383
Age	0.022	0.025	0.856	0.392	1.022	0.973	1.075
Male	1.858	0.478	3.885	<0.001	6.408	2.573	17.060
Urban residence	−0.076	0.539	−0.142	0.887	0.927	0.308	2.620
Current smoker	1.455	0.770	1.890	0.059	4.284	1.049	22.910
Former smoker	1.741	0.812	2.143	0.032	5.702	1.275	32.778
Arterial hypertension	1.029	0.589	1.748	0.081	2.799	0.947	9.853
Diabetes mellitus	0.191	0.530	0.361	0.718	1.211	0.409	3.323
COPD	−0.904	0.485	−1.865	0.062	0.405	0.151	1.025

**Table 15:** Clinical and demographic covariates included in the multivariate logistic regression model for M-Xylene exposure and EGFR positivity. Abbreviations: EGFR, epidermal growth factor receptor; OR, odds ratio; CI, confidence interval; SE, standard error; HTA, arterial hypertension; COPD, chronic obstructive pulmonary disease; M-Xylene, meta-xylene.

Variable	B	SE	t	*p*-Value	OR	95% CI Lower	95% CI Upper
Mean M Xilen (μg/m^3^)	0.266	1.755	0.151	0.079	1.304	0.965	1.764
Age	0.036	0.032	1.124	0.261	1.037	0.975	1.106
Male	1.453	0.575	2.529	0.011	4.276	1.419	13.858
Urban residence	−0.686	0.646	−1.061	0.289	0.504	0.126	1.675
Current smoker	1.147	0.855	1.341	0.180	3.148	0.644	19.803
Former smoker	1.709	0.902	1.895	0.058	5.525	1.047	38.455
Arterial hypertension	0.948	0.671	1.413	0.158	2.580	0.750	10.944
Diabetes mellitus	0.123	0.613	0.200	0.841	1.131	0.318	3.630
COPD	−1.087	0.584	−1.862	0.063	0.337	0.100	1.020

## Data Availability

The anonymized dataset supporting the findings of this study has been deposited in the Open Science Framework (OSF) repository and is available through an anonymous view-only link during the peer-review process (https://osf.io/54f2w/overview?view_only=a523ab3dcaea4f5b85b7355d57bc4d6a, accessed on 28 June 2026). Following publication, the repository will remain available in accordance with institutional and ethical requirements.

## Abbreviations

The following abbreviations are used in this manuscript:

AIC	Akaike Information Criterion
ALK	Anaplastic Lymphoma Kinase
CI	Confidence Interval
CO	Carbon Monoxide
COPD	Chronic Obstructive Pulmonary Disease
CRIPMRD	Center for Research and Innovation in Personalized Medicine of Respiratory Diseases
EGFR	Epidermal Growth Factor Receptor
EGFR-TKI	Epidermal Growth Factor Receptor Tyrosine Kinase Inhibitor
GLOBOCAN	Global Cancer Observatory
HTA	Arterial Hypertension
IARC	International Agency for Research on Cancer
KRAS	Kirsten Rat Sarcoma Viral Oncogene Homolog
NO	Nitric Oxide
NO_2_	Nitrogen Dioxide
NOx	Nitrogen Oxides
NSCLC	Non-Small Cell Lung Cancer
O_3_	Ozone
OR	Odds Ratio
PD-1	Programmed Cell Death Protein 1
PD-L1	Programmed Death-Ligand 1
PM2.5	Particulate Matter ≤2.5 μm
PM10	Particulate Matter ≤10 μm
R^2^	Coefficient of Determination (Pseudo-R^2^ in Logistic Models)
SD	Standard Deviation
SO_2_	Sulfur Dioxide
TPS	Tumor Proportion Score
WHO	World Health Organization
OSF	Open Science Framework
NSCLC NOS	Non-Small Cell Lung Cancer, Not Otherwise Specified
